# Non-Enzymatic Detection of Glucose in Neutral Solution Using PBS-Treated Electrodeposited Copper-Nickel Electrodes

**DOI:** 10.3390/bios11110409

**Published:** 2021-10-21

**Authors:** Lindsey Goodnight, Derrick Butler, Tunan Xia, Aida Ebrahimi

**Affiliations:** 1 School of Electrical Engineering and Computer Science, The Pennsylvania State University, University Park, PA 16802, USA; ljg5355@psu.edu (L.G.); djb84@psu.edu (D.B.); tux42@psu.edu (T.X.); 2 Materials Research Institute, The Pennsylvania State University, University Park, PA 16802, USA; 3 Department of Biomedical Engineering, The Pennsylvania State University, University Park, PA 16802, USA

**Keywords:** copper, nickel, electrochemical sensor, neutral solution, glucose, non-enzymatic

## Abstract

Transition metals have been explored extensively for non-enzymatic electrochemical detection of glucose. However, to enable glucose oxidation, the majority of reports require highly alkaline electrolytes which can be damaging to the sensors and hazardous to handle. In this work, we developed a non-enzymatic sensor for detection of glucose in near-neutral solution based on copper-nickel electrodes which are electrochemically modified in phosphate-buffered saline (PBS). Nickel and copper were deposited using chronopotentiometry, followed by a two-step annealing process in air (Step 1: at room temperature and Step 2: at 150 °C) and electrochemical stabilization in PBS. Morphology and chemical composition of the electrodes were characterized using scanning electron microscopy and energy-dispersive X-ray spectroscopy. Cyclic voltammetry was used to measure oxidation reaction of glucose in sodium sulfate (100 mM, pH 6.4). The PBS-Cu-Ni working electrodes enabled detection of glucose with a limit of detection (LOD) of 4.2 nM, a dynamic response from 5 nM to 20 mM, and sensitivity of 5.47 ± 0.45 ${\mu A\;\mathrm{cm}}^{-2}/\log_{10}\left(\mathrm{mole}.L^{-1}\right)$ at an applied potential of 0.2 V. In addition to the ultralow LOD, the sensors are selective toward glucose in the presence of physiologically relevant concentrations of ascorbic acid and uric acid spiked in artificial saliva. The optimized PBS-Cu-Ni electrodes demonstrate better stability after seven days storage in ambient compared to the Cu-Ni electrodes without PBS treatment.

## 1. Introduction

Diabetes is a worldwide health problem and one of the leading causes of death and disability [1,2]. As such, patients with diabetes are clinically advised to monitor their glucose levels regularly [2,3]. Treatment for diabetes requires accurate glucose monitoring, which has made developing glucose sensors a highly active research area in the biosensor community, especially in the point-of-care testing domain. Conventional electrochemical glucose sensors use the glucose oxidase (GOx) enzyme which offers superior selectivity and good sensitivity for detecting glucose in physiological pH. However, enzymes suffer from stability issues due to their sensitivity to changes of pH, temperature, humidity, and interference of some electro-oxidizable species [4,5,6,7,8,9,10]. Moreover, enzymatic sensors are limited by enzyme leaching, electrode replacement [11], and are not amenable for electrode miniaturization [12].

To address these challenges, non-enzymatic glucose sensors have been developed based on the electro-oxidation of glucose, which can be detected optically or electrochemically. Optical glucose sensors sometimes need labels as in the case of fluorescence-based sensors. Commonly-used fluorescent markers, based on organic dyes [13,14,15] and semiconductor quantum dots [16,17,18], can exhibit photobleaching and toxicity effects. Optical spectroscopic techniques have also been developed, at the cost of being more expensive and difficult to miniaturize [10]. In comparison, non-enzymatic electrochemical sensors offer simpler operation, fast response, ease of miniaturization and scalability, lower cost, low power requirement, and portability [19,20].

Most electrochemical non-enzymatic glucose sensors are based on noble metals and their alloys (e.g., Pt, Au, Pd, and Rh) [21,22,23,24], transition metals (e.g., Cu, Ni, Zn, and Mn) [25,26], metal-oxide (e.g., NiO and CuO) [27,28], semiconductor nanostructures (e.g., graphene and MoS_2_) [11,29], and their combination [6,11,25]. Among various catalytic materials for oxidation of glucose, Ni and Cu compounds are promising due to their low cost, good electrochemical stability, and exceptional electrocatalytic properties [30,31,32,33,34,35,36,37]. For example, decorating Ni nanoparticles (NPs) on glassy carbon electrodes modified with carbon nanotubes exhibited good electrocatalytic activity with a 10 nM limit of detection (LOD) and a wide linear range (0.25–1200 µM) toward the electro-oxidation of glucose in an alkaline NaOH solution [38]. In another work, it was shown that a Cu electrode modified with Ni NPs and multiwalled carbon nanotubes can achieve a 2 µM–10 mM linear range in an alkaline environment [5]. Recent reports of nickel-oxide (NiO) electrodes have also shown a good response for glucose with an advantage of being more stable compared to Ni [6,37,39]. It was shown that NiO electrodes can enable the detection of glucose in the concentration range of 0.005–5.5 mM in NaOH (pH 13) [39]. Ni was first electrodeposited onto nickel foam, followed by annealing at 300 °C for 3 h to fully transform α-Ni(OH)_2_ to NiO [39]. Nafion-coated Cu nanowires synthesized using wet chemistry—using Cu(NO_3_)_2_, concentrated NaOH, EDA, and N_2_H_4_—enabled detecting glucose as low as 35 nM in NaOH (pH 13) with a linear response up to 3 mM [25]. In another work, Cu-Ni modified pencil graphite was used for detection of glucose. Cu was electrodeposited using cyclic voltammetry (CV) onto a pencil graphite in an acidic solution containing copper sulfate (pH 3.5) [40], followed by Ni electrodeposition in a solution containing nickel sulfate (pH 6.5) [41]. The electrode showed a LOD ~ 1 nM in NaOH (pH 13) [40]. There are also reports of non-enzymatic sensors based on precious metals that can detect glucose in neutral solution. For example, in one report, a conventional Au disk was used to detect glucose in a phosphate buffer solution (PBS, pH = 7.40). The linear range was 0.5–20 mM with a LOD of 10 mM [42]. Core-shell structure Au@Pt NPs were also fabricated using a sonochemical method. The At@Pt NPs were found to have a wide linear response (0.5–10.0 μM and 0.01–10.0 mM) in phosphate-buffered saline (pH = 7.4) [24]. Additionally, a report of nanocomposite of Pt:Au on activated carbon (PtAu/C) showed a linear range of 1–20 mM with a LOD of 2 μM in PBS (pH = 7.4) [43]. The PtAu/C nanocomposites were fabricated by suspending and refluxing precursors in a synthetic solution with activated carbon. In another study, Pt/CuO/Pt metal-oxide-metal was fabricated using a wafer-style method. This sensor had a linear sensing range of 2.2 mM-10 mM in pH = 7 [23]. 

However, as highlighted above, non-enzymatic and non-noble metal-based catalytic electrodes for glucose oxidation mostly operate in highly-alkaline media which can be challenging for real-world applications, damage electrode materials, and impose storage/operational hazards [10,32,33,34,35,36]. In this work, we introduce a low-cost and scalable method to synthesize a non-noble metal catalyst for detection of glucose in near-neutral solution. The sensing electrodes are created using sequential electrodeposition of Cu nanostructures on Ni followed by a two-step annealing process—which is optimized—and electrochemical treatment/stabilization in phosphate buffer saline (PBS). The electrochemical sensors using PBS-Cu-Ni as the working electrode show excellent analytical performance with a LOD of 4.2 nM, a dynamic range from 5 nM to 20 mM, and sensitivity of 5.47 ± 0.45 ${\mu A\;\mathrm{cm}}^{-2}/\log_{10}\left(\mathrm{mole}.L^{-1}\right)$ at an applied potential of 0.2 V. The effect of electrodeposition, annealing, and PBS stabilization on surface morphology and elemental composition are studied using scanning electron microscopy (SEM) and energy-dispersive X-ray spectroscopy (EDS). Moreover, the sensors enable selective detection of glucose in a mixture with ascorbic acid and uric acid in diluted artificial saliva. Not only is the PBS treatment critical for the enhanced sensitivity of PBS-Cu-Ni electrodes, but the treatment also improves the sensor stability. 

## 2. Materials and Methods

### 2.1. Chemical Reagents

Analytical grade D-(+)-glucose, L-ascorbic acid, uric acid, and dopamine hydrochloride powders, PBS, sodium sulfate (Na_2_SO_4_), copper sulfate (CuSO_4_) were purchased from Sigma-Aldrich (St. Louis, MO, USA). All the solutions used for electrochemical deposition and sensor testing were prepared using MilliQ Ultrapure deionized (DI) water (18.2 MΩ·cm). 

### 2.2. Electrodeposition of Nickel (Ni) and Copper (Cu)

Glass slides are first cleaned by sonicating sequentially in acetone and isopropanol for 10 min each, followed by rinsing in DI water and drying with N_2_. This is followed by an electron-beam (e-beam) deposition of a seed layer of Cr/Au (10 nm/100 nm). Ni is then electrodeposited in High-Speed Nickel Sulfamate Plating Solution (Technic Co., Cranston, RI, USA) using a two-electrode configuration, comprised of a stainless-steel reference/counter electrode. Ni of ~10 µm thickness is deposited using chronopotentiometry with a current density of −6 mA/cm^2^ for 125 min. After rinsing the substrate with DI water, Cu is electrodeposited via chronopotentiometry at a current density of −3 mA/cm^2^ for 2 min (in the same setup used for Ni deposition). The deposition solution consists of 5 mM of CuSO_4_ as the Cu source mixed in 50 mM of Na_2_SO_4_ as the background electrolyte (pH 6.4). The PalmSens4 potentiostat and PSTrace5 software (BASi Co., West Lafayette, IN, USA) are used to control the electrodeposition processes.

### 2.3. Scanning Electron Microscopy (SEM) and Energy-Dispersive X-ray Spectroscopy (EDS)

SEM images are captured using a FEI Verios G4 instrument, using a working distance of ~3 mm. For the EDS measurements, a beam energy of 10 kV is used to ensure all necessary elements can be detected. 

### 2.4. X-ray Photoelectron Spectrscopy (XPS) Measurements

XPS measurements are performed in a Physical Electronics VersaProbe II instrument with an Al Kα source (1.49 keV) and charge neutralization. A takeoff angle of 45° is used. Data are analyzed using the CasaXPS software.

### 2.5. Electrochemical Characterization

Electrochemical characterization is carried out using cyclic voltammetry (CV). A three-electrode configuration with a platinum counter electrode (BASi Co., Indiana, USA), Ag/AgCl reference electrode (BASi Co., Indiana, USA), and the developed electrodes as the working electrode is employed. The electrochemical measurement setup is contained in an electrical probe station. To prepare the samples for testing the sensors, glucose powder is dissolved in a solution of 100 mM Na_2_SO_4_ in DI water at different concentrations. CV measurements are used to evaluate the response to different glucose concentrations as well as interfering analytes. Each CV cycle consists of a reduction direction from 0.35 to −0.6 V, followed by oxidation in the opposite direction. CV scan rate of 50 mV/s is used for analytical testing. In all electrochemical measurements, polyimide tape is used to isolate the active electrode area (d = 2 mm) from the contact pads and electrical probes.

## 3. Results and Discussion

### 3.1. Sensor Development and Characterization 

**Figure 1:**
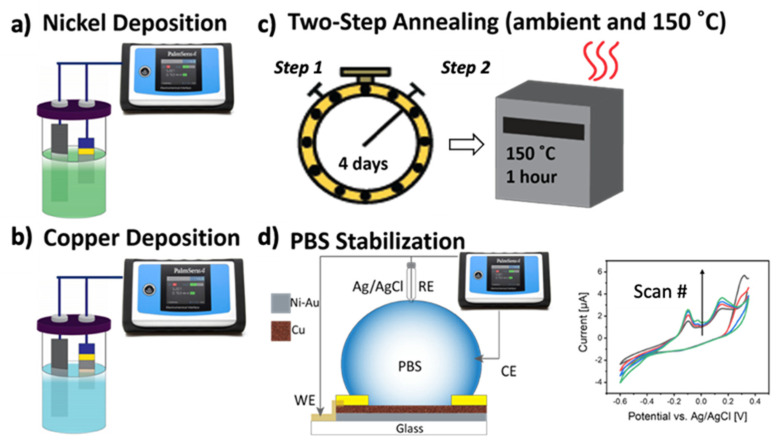
Schematic for fabrication of phosphate-buffered saline (PBS)-Cu-Ni working electrodes. (**a**) Electrodeposition of nickel (Ni) followed by (**b**) copper (Cu) electrodeposition at 50 °C for 2 min. (**c**) The two-step annealing process, with Step 1 in air at room-temperature for four days, followed by Step 2 at 150 °C for 1 h. (**d**) Electrochemical treatment of Cu-Ni electrodes in PBS (pH = 7.4) using cyclic voltammetry (CV) with a scan rate of 50 mV s^−1^. At least 45 scans are performed until CV curves are stabilized. The inset shows representative CV curves.

Figure 1 demonstrates the process for synthesis of PBS-Cu-Ni electrodes. Briefly, a ~10 µm Ni layer is first electrodeposited at a constant current density of −6 mA/cm^2^ for 125 min on thin Au films on glass substrates. Subsequently, Cu is electrodeposited onto the Ni surface at a constant current density of −3 mA/cm^2^ for 2 min. After storing the sample in ambient condition for 4 days (Step 1), the Cu-Ni electrode is further annealed at 150 °C for 1 h (Step 2). We investigated various annealing conditions (summarized as T1, T2, T2P, and T3 in the Supplementary Information, SI, Figure S1a) and showed that the initial long ambient annealing (4 days) is required to achieve high sensitivity (compare T1:4-day ambient annealing and T3:1-day ambient annealing in SI, Figure S1). We believe the initial prolonged stabilization/annealing in an air step creates stable phases of copper oxide which is shown to demonstrate good catalytic properties for oxidation of glucose [33].

After the two-step annealing process, we create PBS-Cu-Ni electrodes by performing cyclic voltammetry (CV) in PBS until stabilization of CV curves (i.e., overlap of successive CV curves). Our results in Figure S1b comparing different annealing conditions suggest that without PBS treatment, the sensor response is poor (timeline T2). PBS treatment not only enhances the sensor sensitivity toward glucose (compare T2 with T2P in SI, Figure S1b), but also significantly improves its stability (discussed later). 

**Figure 2:**
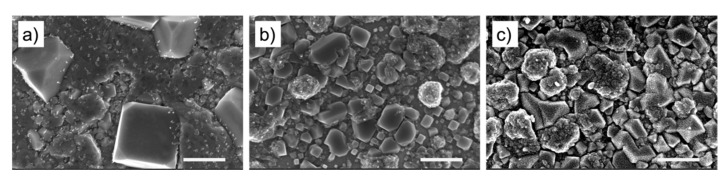
Scanning electron microscope (SEM) images of (**a**) Cu-Ni electrode before the two-step annealing process, (**b**) Cu-Ni electrode after the two-step annealing, (**c**) PBS-treated Cu-Ni (PBS-Cu-Ni) electrode. Scale bar: 500 nm.

We studied the effect of annealing and PBS treatment on surface morphology. Comparing the SEM images in Figure 2a,b suggest that annealing results in growth of Cu crystal nucleation sites as well as improving the surface uniformity. After electrochemical stabilization/treatment in PBS, the surface morphology undergoes further changes—crystal growth and formation of additional nanostructures on top of the Cu microcrystals—as shown in Figure 2c. It should be noted that electrodeposition of Cu results in the formation of a hierarchical layer of Cu nanostructures covering the Cu microstructures on the Ni layer (see SEM images in SI, Figure S2). X-ray photoelectron spectroscopy (XPS) spectra of Cu-Ni samples shown in SI, Figure S3, suggest that copper is mainly oxidized to CuO.

**Figure 3:**
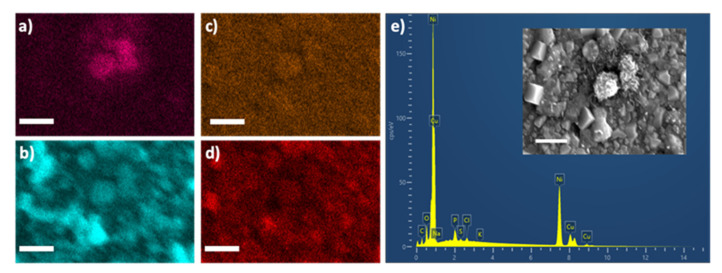
Morphology and elemental composition of PBS-Cu-Ni electrode. Energy-dispersive X-ray spectroscopy (EDS) maps for (**a**) chlorine (Cl), (**b**) copper (Cu), (**c**) phosphorous (P), (**d**) oxygen (O). (**e**) The corresponding EDS spectra. Inset—SEM image of the mapped region. Scale bar: 1 µm.

Figure 3a–d depict the EDS maps for chlorine, copper, phosphate, and oxygen respectively in a PBS-Cu-Ni electrode. Figure 3e demonstrates the EDS spectrum with the corresponding SEM image (inset) indicating the formation of nanostructures. The nanostructures in PBS-Cu-Ni electrodes are speculated to enhance the sensing capability by providing a high surface area and more active sites to facilitate the electro-catalysis of glucose. Increase of the active sites results in improved sensing [44]. Furthermore, modification of electrodes with nano- and micro-particles has been reported to affect the apparent reaction kinetics, even if the diffusion layer extends beyond the scale of the electrode surface features [45]. Interestingly, flower-like nanostructures of Cu_3_(PO_4_)_2_ were reported to form spontaneously by exposing copper to PBS, which resulted in improved protein functionalization by He et al. [46]. 

The EDS maps in Figure 3 indicate that after PBS treatment, Cl is present on the copper surface which is similar to the previous reports [46], where the authors showed that the Cl^−^ ions in PBS are indispensable for the formation of the nanoflowers. It was observed that the flower-like structures did not form if Cl^−^ was not present in the solution during material synthesis [46]. Because of the similarity of our work with the work of He et al., we speculate that morphology changes after PBS treatment is caused by dissolved oxygen, Cl^−^, and PO_4_^3−^ [46]. It is proposed that in the presence of Cl^−^ ions, Cu is oxidized by the dissolved oxygen and forms CuCl (corresponding to oxidation peak at −0.2 V in Figure 1d), which in turn converts to CuCl^2−^, followed by dissolving with phosphate to form Cu_3_(PO_4_)_2_. Another possibility is formation of Cu_2_O via hydrolysis via the following reaction [46].

2CuCl + 2OH^−^ → 2Cl^−^ + Cu_2_O + H_2_O (1)

The oxidation peak at ~0.2 V in Figure 1d can be associated to a combination of Cu(0)/Cu(II) and Cu(I)/Cu(II) redox couples (e.g., through oxidation of Cu_2_O to CuO) [25,47].

**Figure 4:**
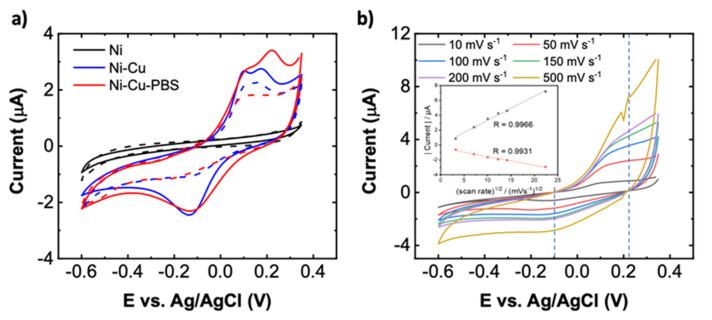
(**a**) Cyclic voltammogram (CV) at 0 (dashed lines) and 5 mM (solid lines) glucose in 0.1 M Na_2_SO_4_ for Ni, Cu-Ni, and PBS-Cu-Ni electrodes. The scan rate of 50 mV/s is used. (**b**) CV curves at different scan rates from 10 to 500 mVs−^1^ with 1 mM glucose in 0.1 M Na_2_SO_4_ with a PBS-Cu-Ni electrode. The inset shows the relationship between the oxidation (black curve) and reduction (red curve) current at 0.22 V and −0.1 V (indicated by vertical dashed lines), respectively, vs. the square root of the scan rate.

**Figure 5:**
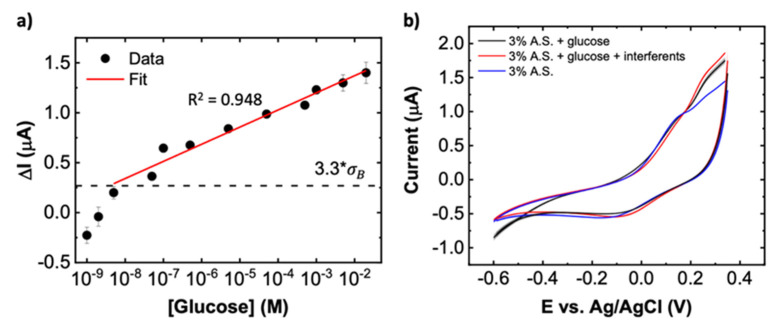
Sensor sensitivity, selectivity, and response in artificial saliva. (**a**) Baseline-subtracted values of oxidation current at 0.21 V for various glucose concentrations in 0.1 M Na_2_SO_4_ (pH 6.4). The error bars depict the standard errors with n = 6–9. $\sigma_{B}$ represents the standard deviation of the blank solutions. (**b**) Average CV response in 3% diluted artificial saliva (A.S.) with either no glucose or spiked with 65 μM glucose or 65 μM glucose with interferents consisting of a mixture of UA (175 μM) and AA (600 nM) spiked into artificial saliva before dilution of solutions. The curves are obtained from an average of 5 CV scans and the shaded regions surrounding the curve represents the standard error of the 5 CV scans. Scan rate = 50 mV s^−1^.

In addition to material characterization, we evaluated the characteristics of electrodes before and after deposition of Cu and after PBS treatment using CV measurements over a range of −0.6 V to +0.35 V vs. Ag/AgCl reference electrode. Figure 4a displays the cyclic voltammograms of PBS-Cu-Ni (one of the sensors used in calculating the calibration curve in Figure 5a), Cu-Ni, and Ni electrodes in 0.1 M Na_2_SO_4_ in the presence of 5 mM glucose at a scan rate of 50 mVs^−1^. These results confirm that deposition of Cu increases the oxidation current in the presence of glucose compared to bare Ni ($I_{\mathrm{Ni}}=0.439\;\mu A\;\mathrm{vs}.{\;I}_{\mathrm{Ni}-\mathrm{Cu}}=2.53\;\mu A\;$at the sensing potential of 0.21 V), noting that bare Ni is not catalyzing glucose oxidation as there is no Faradaic peak in the CV curve. Importantly, PBS treatment of the Cu-Ni electrodes results in an additional current increase ($I_{\mathrm{PBS}-\mathrm{Cu}-\mathrm{Ni}}=3.38\;\mu A$). The presence of two oxidation peaks for Cu-containing samples are speculated to be due to the Cu(0)/Cu(II) and Cu(I)/Cu(II) redox couples, which often occur at similar potentials [25,47]. A rough mechanism for oxidation of glucose at PBS-Cu-Ni electrode is expected to be the following: Cu_3_(PO_4_)_2_ or Cu_2_O will electrochemically oxidize to Cu(III) species such as CuOOH or Cu(OH)_4_. Next, glucose is oxidized by Cu(III) to form hydrolyzate gluconic acid,

Cu(III) + glucose → gluconolactone + Cu(II),

followed by gluconolactone → gluconic acid conversion *via* hydrolysis [40]. We believe that electro-oxidation of glucose to gluconolactone may also be catalyzed by the Ni(II)/Ni(III) redox couple, [40] although the dominant contribution is through copper redox reactions as evident from significantly smaller Faradaic current with bare Ni electrode vs. Cu-Ni (Figure 4a).

Figure 4b shows the CV of PBS-Cu-Ni electrodes recorded in 0.1 M Na_2_SO_4_ solution at different scan rates in the range of 10–500 mVs^−1^. It is found that the anodic and cathodic currents at ~0.2 V and −0.1 V, respectively, are linearly correlated to the square root of the scan rate with a linear regression of $I_{\mathrm{pa}}\;\left[\mu A\right]=-0.07685+0.32479x\;\left(R=0.993\right)$ and $I_{\mathrm{pc}}\;\left[\mu A\right]=-0.39673-0.1179x\;\left(R=0.996\right)$, respectively. The linear dependence on the square root of scan rate indicates that the glucose redox reaction is a diffusion-controlled process based on the Randles-Ševčík equation [48].

### 3.2. Sensor Performance: Sensitivity, Selectivity, Response in Artiificial Saliva, and Stability

As previously discussed in the Introduction Section, Cu and Ni have been extensively utilized in developing non-enzymatic sensors for detection of glucose oxidation [26,49,50,51]. However, much of the prior reports require operating in highly alkaline solutions or require at least 0.5 or 0.6 V to achieve large signal to noise. In contrast, our results using PBS-treated Cu-Ni electrodes (Figure 5a) show that glucose can be detected in an outstanding wide range from 5 nM to 20 mM at near-neutral pH of 6.4 using only ~0.2 V vs. Ag/AgCl. The signal is defined as,

(2) $$\Delta I=I_{E=0.21\;V}-I^{0}{}_{\mathrm{avg},\;E=0.21\;V}$$

where $I_{E=0.21\;V}$ is the current at E = 0.21 V for a given sample, glucose concentration (ρ), and CV scan number, and $I^{0}{}_{\mathrm{avg},\;E=0.21\;V}$ is the average baseline (without glucose) obtained for each sample using 7 separate CV scans. The data in Figure 5a are fitted (R^2^ = 0.948) using the following equation,

(3) $$\Delta I\;\left[\mu A\right]=1.71+0.172*\log_{10}\left(\rho \;\left[M\right]\right)$$

From the fit, the sensitivity of the proposed sensor is found to be 5.47 ± 0.45 ${\mu A\;\mathrm{cm}}^{-2}/\log_{10}\left(\mathrm{mole}.L^{-1}\right)$ (the uncertainty represents standard error of the fit). We calculate a limit of detection of LOD = 4.2 nM at 3.3 times the standard deviation of the blank solution, $\sigma_{B}$ (dashed black line in Figure 5a; see SI for more details on the LOD calculation). As a more conservative estimate, one can also define the limit of quantitation as 10$\sigma_{B}$, yielding a value of 6.7 μM [52,53]. The ultralow detection capability is believed to stem from the combination of Cu, Cu_3_(PO_4_)_2_, and Cu_2_O, which is formed as a result of the CV treatment in PBS and reduction of CuO to Cu_2_O. All three materials have previously been used for glucose detection and have demonstrated exceptional performance [54,55,56,57].

In addition to high sensitivity and low detection limit, a good sensor should also be selective toward the target analyte. A main challenge of non-enzymatic glucose sensors is the interference of other substances besides glucose, which can be oxidized at potentials comparable to glucose. Biomolecules, including ascorbic acid (AA) and uric acid (UA) often co-exist with glucose in human biofluids, and hence are important interferents to consider in the electrochemical oxidation of glucose. In physiological samples of saliva, the glucose concentration is around 65 μM, UA is 175 μM, and AA is 600 nM. In order to study the sensor performance for selective quantification of glucose in a biological sample, we tested PBS-Cu-Ni sensors with artificial saliva (A.S.) spiked with analytes with these physiological concentrations. Before testing, the samples are diluted to 3% in 0.1 M Na_2_SO_4_. As shown in Figure 5b, the average CV responses with glucose and with glucose + interferents (i.e., UA and AA) show an increase in current compared with the baseline. The CV curve for glucose alone is similar to that of glucose + interferents, indicating AA and UA have only a minor effect on the glucose sensing performance in A.S. We should note, however, that the signal seen in artificial saliva (ΔI~0.25 μA at the peak potential) is lower in magnitude than what is expected based on the calibration curve of Figure 5a (ΔI~0.7 μA) which is in Na_2_SO_4_. The peak is also shifted to a more positive potential (compare Figure 4a with Figure 5b). This is most likely a result of biofouling effects from interferents in the artificial saliva that can hinder the response to glucose. Extending to applications with real saliva samples, future work could explore surface protection materials, such as Nafion, to minimize the fouling effect and maintain a high level of performance in complex sample matrices.

**Figure 6:**
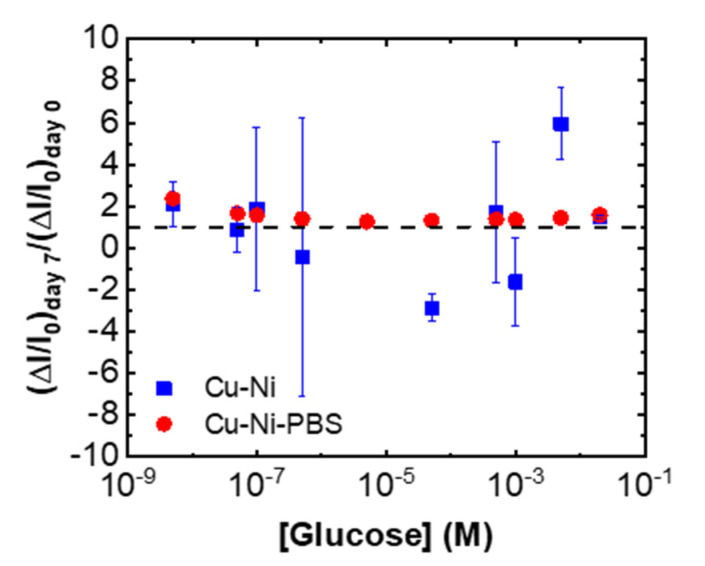
PBS treatment is essential for sensor stability. The oxidation current (ΔI/I_0_) on day 7 normalized to the initial signal (on day 0) over the entire sensor dynamic range (5 nM–20 mM) at a potential of 0.21 V. The sample was stored in ambient condition. Error bars represent standard error of 3 CV scans from 2–3 distinct sensors. The dashed line represents the ideal sensor stability, i.e., when day 7 signal equals that of day 0.

The long-term stability is another important factor when comparing different sensors. We measured the response of Cu-Ni and PBS-Cu-Ni after 1 week with different concentrations of glucose in 0.1 M Na_2_SO_4_. The normalized signals (calculated based on the oxidation current at 0.21 V; details are provided in SI) with respect to the first day are plotted in Figure 6, suggesting a significantly larger variability of Cu-Ni samples compared to PBS-Cu-Ni samples after 7 days of ambient storage. These results demonstrate the importance of the PBS treatment for improving sensor stability.

Table 1 provides a comparison between some of the reported non-enzymatic glucose sensors with our work. Except a few works which utilize noble metals (such as Au and Pt) [10,23,24], majority of non-enzymatic glucose sensors require alkaline pH. In comparison, the developed sensing material can operate in a neutral solution which offers safe storage of reagents and operation, less environmental impact, and minimized damage/corrosion of the sensor electrodes.

## 4. Conclusions

This work demonstrates the synthesis and application of PBS-treated Cu-Ni electrodes as the working electrode for non-enzymatic electrochemical sensing of glucose in near-neutral solution, which is a distinct advantage compared to other non-noble metal glucose catalysts. PBS-Cu-Ni electrodes show selective response to glucose in the presence of uric acid and ascorbic acid in artificial saliva. Cyclic voltammetry analysis showed an ultralow LOD of 4.2 nM, a wide dynamic response from 5 nM to 20 mM, and a sensitivity of 5.47 ± 0.45 ${\mu A\;\mathrm{cm}}^{-2}/\log_{10}\left(\mathrm{mole}.L^{-1}\right).$ In addition to enhancing the sensor sensitivity, PBS treatment is essential for stable operation in the entire dynamic concentration range. With further material optimization and coating with antifouling layers, the proposed all-electrochemically synthesized electrodes may offer the ability to directly measure glucose in more biologically complex media, such as blood. Electrodeposition and low-temperature processing are ideal for selective functionalization of the electrode array for further reducing sensor cost as well as developing sensors on flexible substrates such as paper and plastic.

## Figures and Tables

**Table 1:** A comparison between this work and the literature regarding non-enzymatic glucose detection.

Material	Dynamic Range	Limit of Detection	Applied Potential	Medium pH	Sensitivity	Reference
Ni NPs on GCE modified with CNTs	0.1–5000 µM	2 nM	0.4 V	13	0.0025 mA mM^−1^	[38]
Ni NPs on GCE modified with CNTs	2 μM–10 mM	0.7 µM	0.35 V	13	3.8 mA mM^−1^ cm^−2^	[5]
NiO	0.005–5.5 mM	5 µM	0.47 V	13	6657.5 mA mM^−1^ cm^−2^	[39]
Cu NWs	35 nM–3 mM	35 nM	0.6 V	13	420.3 μA cm^−2^ mM^−1^	[25]
Ni-Cu/PGE	1 nM–10 mM	1 nM	0.5 V	13	2951 μA mM^−1^ cm^−2^	[40]
Cu/MWCNT	0.7 μM–3.5 mM	0.21 μM	0.65 V	12	17.76 μA mM^−1^	[58]
Cu/SWCNT/GCE	0.5–100 μM	0.25 μM	0.65 V	12.7	256 ± 3 μA mM^−1^	[59]
Ni-Cu/TiO_2_ NTs	10 μM–3.2 mM	5 μM	0.6 V	13	1590.9 μA mM^−1^ cm^−2^	[49]
Cu_x_O/PPy/Au	6.2 μM–8 mM	6.2 μM	0.6 V	13	232.22 μA mM^−1^ cm^−2^	[60]
Pt/CuO/Pt	2.2 mM–10 mM	2.2 mM	1 V	7	2921 μA mM^−1^cm^−2^	[23]
Au@Pt NPs	0.5–10.0 μM and 0.01–10 mM	445 nM	0.1 V and 0.35 V	7.4	0.5755 μA mM^−1^	[24]
Au disk	0.5–20 mM	10 μM	0.25 V	7.4	0.72 mA mM^−1^ cm^−2^	[42]
PtAu/C	0–10 mM	2 μM	0.35 V	7.4	4.7 μA mM^−1^ cm^−2^	[43]
PBS-Cu-Ni	5 nM–20 mM	4.2 nM	0.2 V	6.4	5.47 μA cm^−2^/log_10_ (M^−1^)	This work

NPs: nanoparticles, CNTs: carbon nanotubes, GCE: glassy carbon electrode, MWCNTs: multi-walled carbon nanotubes, PGE: Pencil graphene electrode, SWCNT: single-walled carbon nanotube, PPy: polypyrrole.

## Data Availability

Data is contained within the article or supplementary material. The raw data presented in this study are available upon request.

## Supplementary Materials

The following are available online at https://www.mdpi.com/article/10.3390/bios11110409/s1, Figure S1. (**a**) Various annealing conditions used for optimization of the glucose sensor. “Day 11 testing” was performed to evaluate the sensor stability (referred as day 7 after initial testing in the main text). “Annealing” refers to ambient annealing at 150°C for one hour. (**b**) The signals resulting from the various annealing conditions. Figure S2. SEM images of (**a**) bare Ni, (**b**) Cu nanostructures deposited on Ni surface. Scale bar: 10 μm. Figure S3. X-ray photoelectron spectra (XPS) for Cu 2p. Based on the position of the peaks, it is determined that Cu is mainly oxidized as CuO.
