# Supplementary material for: Non-Enzymatic Detection of Glucose in Neutral Solution Using PBS-Treated Electrodeposited Copper-Nickel Electrodes
Source: Biosensors (Basel). 2021 Oct 21;11(11):409. doi: 10.3390/bios11110409 (PMC8615574; doi:10.3390/bios11110409)
Supplement: Supplementary file 1 [file biosensors-11-00409-s001.zip › biosensors-1363972-supplementary-for Conversion.pdf]

biosensors

Supplementary

Non-Enzymatic Detection of Glucose in Neutral Solution Using PBS-Treated Electrodeposited Copper-Nickel Electrodes

Lindsey Goodnight [1], Derrick Butler [1,2], Tunan Xia [1] and Aida Ebrahimi [1,2,3,*]

[1] School of Electrical Engineering and Computer Science, The Pennsylvania State University, University Park, PA 16802, USA; ljg5355@psu.edu (L.G.); djb84@psu.edu (D.B.); tux42@psu.edu (T.X.)
[2] Materials Research Institute, The Pennsylvania State University, University Park, PA 16802, USA
[3] Department of Biomedical Engineering, The Pennsylvania State University, University Park, PA 16802, USA
* Correspondence: sue66@psu.edu

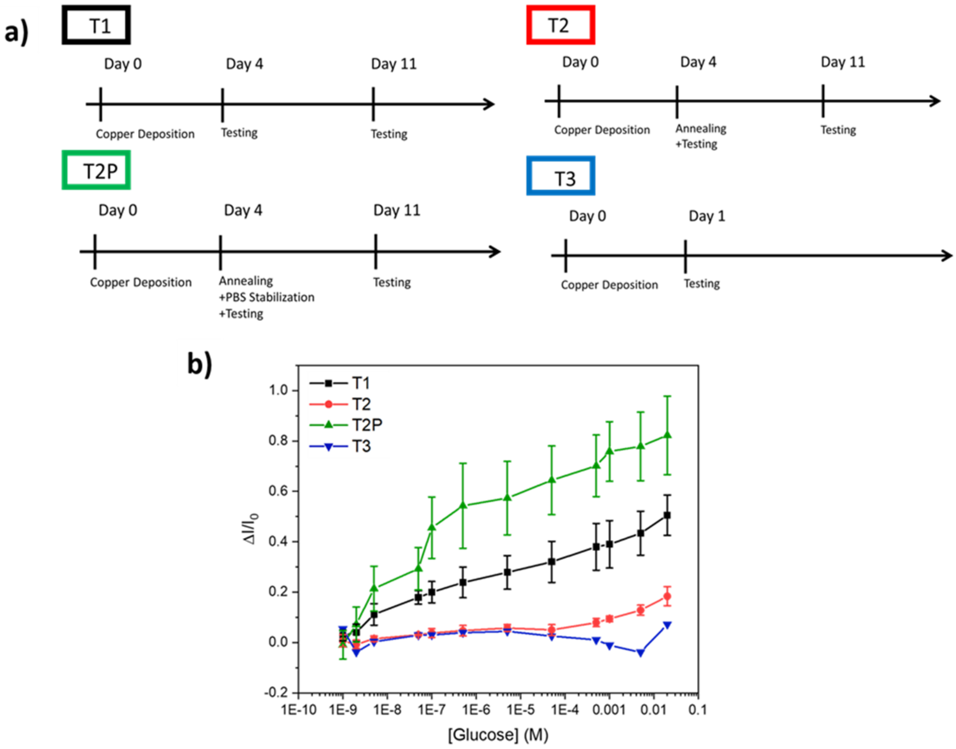

Figure S1. (**a**) Various annealing conditions used for optimization of the glucose sensor. “Day 11 testing” was performed to evaluate the sensor stability (referred to as day 7 after initial testing in the main text). “Annealing” refers to ambient annealing at 150°C for one hour. (**b**) The signals resulting from the various annealing conditions.

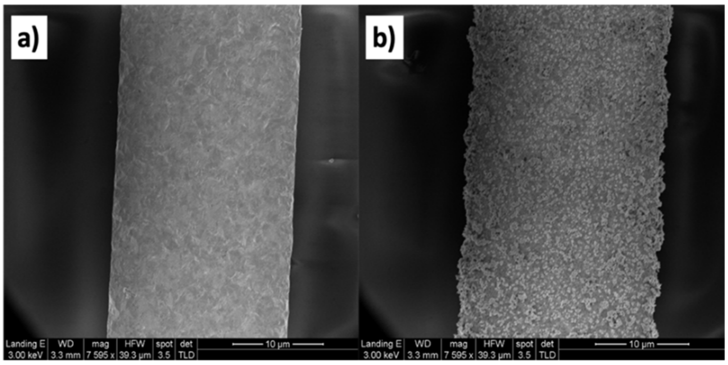

Figure S2. SEM images of (**a**) bare Ni, and (**b**) Cu nanostructures deposited on Ni surface. Scale bar: 10 μm.

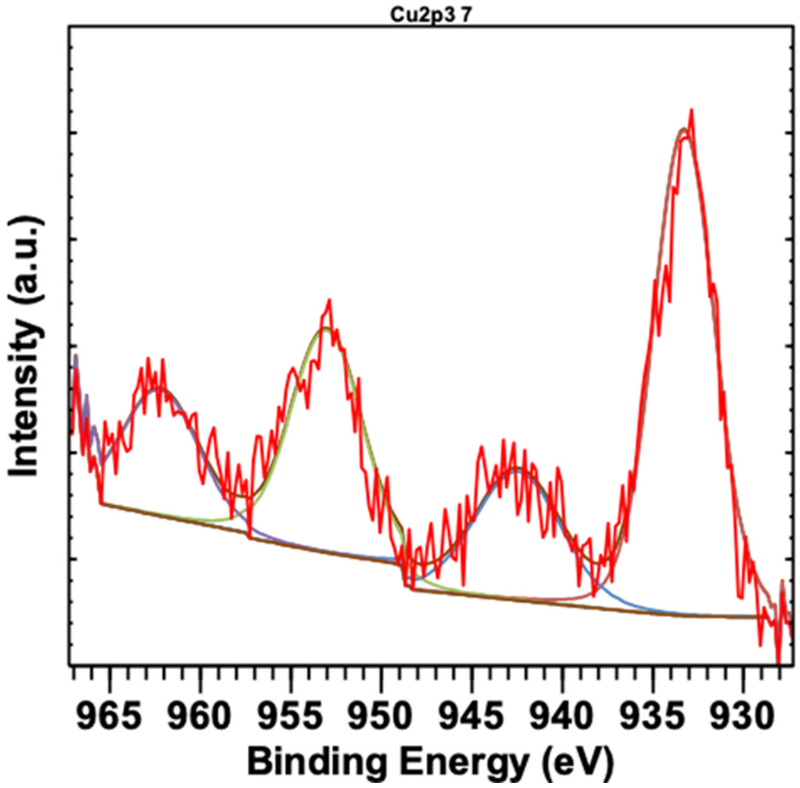

Figure S3. X-ray photoelectron spectra (XPS) for Cu 2p. Based on the position of the peaks, it is determined that Cu is mainly oxidized as CuO.

Limit of detection calculation:

Each data point in Figure 5a of the main text contains contributions from 3 distinct sensors. First, the average baseline ($I_{0,\ avg.}$), i.e., 0.1 M Na_2SO_4 without glucose, is calculated for each sensor using 3 distinct CV scans after the sample is stabilized. "Stabilized" means the current CV curve traces the previous scan very closely and no apparent shift in the voltammogram features is observed. With $I_{0,\ avg.}$ calculated at a potential of 0.21 V for each sensor, we then subtract the corresponding $I_{0,\ avg.}$ from the peak current (I) at each glucose concentration for each sensor,

$$\Delta I = I_{glucose,sensor\ i} - I_{0,avg,sensor\ i} \qquad (1)$$

where $i = 1, 2, or\ 3$ corresponding to the 3 distinct sensors tested. For each sensor at each concentration of glucose, 3 CV scans are recorded. The data in Figure 5a represent the mean and standard error of ΔI at each glucose concentration. The data are then fitted to obtain a slope (i.e., sensitivity) and intercept. In Figure 5a, the fitting equation (R^2= 0.948) is,

$$\Delta I\ [\mu A] = 1.71 + 0.172 * \log_{10}(\rho\ [M]) \qquad (2)$$

where ρ is the glucose concentration. For the fitting, we only include concentrations with an average $\Delta I > 0$. This limits the input data to 5 nM and above.

To obtain a limit of detection (LOD), we first calculate the standard deviation of the blank solutions (σ_B) as described above. The average of 7 blank CV scans is calculated for each distinct sensor. In this case, "blank" refers to the 0.1 M Na_2SO_4 without any glucose added. The corresponding average values are subtracted from each individual blank CV scan for a given sensor. This is carried out for each of the 3 distinct sensors, yielding 21 blank measurements. Seven scans are included for each sensor to ensure the number of blank measurements is > 20 [1]. The average value of these 21 measurements is ~ 0 (9.5E-7) and the standard deviation σ_B is 0.082 μA. The LOD is calculated as the glucose concentration at the intersection of the fit (Eq. 2) and the line $\Delta I = 3.3 * \sigma_B$.[2, 3]. In other words,

$$3.3 * 0.082 = 1.71 + 0.172 * \log_{10}(\rho_{LOD}\ [M]) \quad (3)$$

where ρ_{LOD} is the LOD. From here, we obtain a LOD = 4.2 nM. As a more conservative estimate, we also calculate the limit of quantitation (LOQ). The LOQ is commonly defined as 10 times the standard deviation of the blanks ($10 * \sigma_B$). Using the same approach looking at the intersection of the calibration curve and the line $\Delta I = 10 * \sigma_B$, we obtain a LOQ of 6.7 μM.

Stability calculation:

The data points in Figure 6 are calculated from 3 scans at each glucose concentration at a potential of 0.21 V. First, the average baseline $I_{0,avg}$ (0.1 M Na_2SO_4, [glucose] = 0) is calculated from 3 distinct CV scans for each sensor (3 sensors for Cu-Ni, 2 sensors for Cu-Ni-PBS) and each day (0 and 7). Then, $I_{0,avg}$ is used to calculate $\Delta I/I_{0,avg}$ at each concentration (5 nM-20 mM) for each of 3 independent scans. This calculation is carried out for each sensor on day 0 and day 7. After that, an average $\Delta I/I_{0,avg}$ is calculated for day 0 and day 7 for each scan (e.g. $(\Delta I/I_{0,avg})_{\text{day 0, scan 1, Sensors 1-3}}$). These values are then used to calculate the normalized signal for each scan,

$$\frac{\Delta I/I_{0,avg}|_{\text{day 7,scan 1,Sensors 1-3}}}{\Delta I/I_{0,avg}|_{\text{day 0,scan 1,Sensors 1-3}}} \quad (4)$$

Finally, an average normalized signal is calculated for each concentration from scans 1-3 and error bars represent the standard error of this average.

References

1. Long, G.L.; Winefordner J. D. Limit of detection. A closer look at the IUPAC definition. *Anal. Chem* **2008**, 55, 712–724. https://doi.org/10.1021/AC00258A001.

2. Huang, Y.Q.; Zhao, S.H.; Zhang, W.X.; Duan, Q.Y.; Yan, Q.; Fu, H.; Zhong, L; Yi, G. Multifunctional electrochemical biosensor with "tetrahedral tripods" assisted multiple tandem hairpins assembly for ultra-sensitive detection of target DNA. RSC Adv **2021**, *11*, 20046–20056. https://doi.org/10.1039/D1RA02424H.

3. Chen, Y.; Li, Y.L.; Yang, Y.; Wu, F.N.; Cao, J.; Bai, L.J. A polyaniline-reduced graphene oxide nanocomposite as a redox nanoprobe in a voltammetric DNA biosensor for Mycobacterium tuberculosis. Microchim Acta **2017**, *1846*, 1801–1808. https://doi.org/10.1007/S00604-017-2184-5.
